# Selected Elements of Lifestyle and the Level of Functional Fitness in Older Women

**DOI:** 10.3390/ijerph19042066

**Published:** 2022-02-12

**Authors:** Antonina Kaczorowska, Anna Sebastjan, Małgorzata Kołodziej, Małgorzata Fortuna, Zofia Ignasiak

**Affiliations:** 1 Institute of Health Sciences, University of Opole, 45-060 Opole, Poland; 2 Department of Biostructure, Wrocław University of Health and Sport Sciences, 51-612 Wrocław, Poland; anna.sebastjan@awf.wroc.pl (A.S.); malgorzata.kolodziej@awf.wroc.pl (M.K.); zofia.ignasiak@awf.wroc.pl (Z.I.); 3 Faculty of Medical Sciences and Technology, The Karkonosze University of Applied Sciences in Jelenia Góra, 58-503 Jelenia Góra, Poland; malgorzata.fortuna@kpswjg.pl

**Keywords:** ageing, functional fitness, Senior Fitness Test, social welfare homes, community-dwelling elderly

## Abstract

Background: The appropriate level of functional fitness is a very important element for seniors to maintain self-reliance in daily life. The aim of this research was to assess sociodemographic differences, selected elements of lifestyle, and functional fitness in the older residents of social welfare homes and community dwellers Methods: The analysed group comprised 693 women aged 65–79, including 173 subjects living in social welfare homes and 520 community-dwelling women. Basic anthropometric features were measured, and functional fitness was assessed using the Senior Fitness Test. Basic sociodemographic characteristics, as well as data on health self-assessment and selected elements of lifestyle, were also collected. Results: The female residents of social welfare homes were found to have a lower body mass index (BMI), and they came from smaller cities, compared with community-dwelling older women. Furthermore, almost a third of them had no children and completed primary or vocational education. They also reported smoking, poor health conditions, and lack of physical activity. The functional fitness of women living in social welfare homes was significantly lower than in community-dwelling women. Conclusions: As seniors living in social welfare homes have such a significantly reduced level of functional fitness, compared with their peers living independently, it is necessary to include them in adaptive physical activity and diversified daily activities.

## 1. Introduction

The process of ageing is influenced by many factors, such as physical activity, diet, lifestyle, and the use of stimulants. The appropriate level of functional fitness is a very important element for seniors to maintain self-reliance in daily life. Functional fitness is defined as the physiological ability to carry out normal activities of daily living safely, independently, and without excessive fatigue [1]. The main components of functional physical fitness are the strength and flexibility of the lower and upper body, aerobic capacity, motor coordination, and dynamic balance. These elements allow seniors to perform daily activities necessary for self-reliant living, such as housework, walking, climbing stairs, and carrying objects [1]. Maintaining physical fitness at a level sufficient to efficiently carry out everyday activities and to be independent is of great importance for the quality of life of older people [2,3]. 

However, seniors may develop sarcopenia, which is associated with a decrease in muscle mass and strength. It can result in a deterioration of physical fitness, limitations in performing daily activities, and even disability or death. As the presence of sarcopenia increases the risk of falls, fractures, heart and respiratory diseases, it increases the cost of healthcare [4]. A decline in physical fitness and exercise tolerance is physiological and inevitable with age, but the rate of this decrease varies between individuals. Lifestyle significantly affects the health and functional fitness of seniors. Health-promoting behaviours include physical activity and adequate nutrition, while anti-health behaviours are basically related to addictions, smoking cigarettes, and excessive alcohol consumption. Physical activity in the elderly can slow down the rate of decline in physical fitness and endurance, reduce negative health changes, and help them maintain the ability to perform activities of daily living and functioning. It also allows them to maintain walking speed, healthy body composition, cardiovascular and respiratory functions, and posture stability [5,6]. Physical activity also prolongs the period of mental fitness of seniors. Moreover, the level of physical activity varies from person to person, depending on factors, such as age, education, and gender, as well as motivation, support of the loved ones, and awareness of one’s own needs [7]. In addition to the individual characteristics of each person, the level of physical activity is influenced by the social environment—namely, active neighbours, support of local social groups promoting an active lifestyle, place of residence, accessibility of walking and cycling paths, and means of physical activity [8]. The place of residence, in particular, can affect the physical, social, and mental functioning of seniors [9]. 

Exercise programmes, introduced for community-dwelling seniors, may improve the level of their fitness. For example, Furtado et al. studied community-dwelling elderly women and institutionalised women. Each group followed an 8-week multimodal training programme which consisted of three sessions each week. The improvement was found in community-dwelling women based on all trials of the Senior Fitness Test after 8 weeks of training [10]. Other researchers also reported the effectiveness of the 6-week, short-term, high-intensity programme which improved motor functions in community-dwelling older adults [11]. Exercise programmes introduced for institutionalised seniors can also improve the level of their physical fitness. Some scientists found that 12 weeks of resistance and multicomponent training are effective in increasing functional fitness in institutionalised older women [12]. In addition, the non-exercising institutionalised women showed a fairly rapid decline in fitness levels [12]. Johnen and Scott studied elderly institutionalised women and men as well. They found that free weight training and machine training were both effective in improving fitness [13] contrary to Furtado et al., who reported no improvement after 8 weeks of training in institutionalised women [10].

The aim of the research was to assess sociodemographic differences, selected elements of lifestyle, and functional fitness in the female residents of social welfare homes and community-dwelling women.

## 2. Materials and Methods

### 2.1. Subjects

The research was conducted among female residents of social welfare homes (SWH). The subjects volunteered for free tests thanks to invitations addressed to the centres associating the elderly. They were sent to 15 social welfare homes, and managers of all institutions approved of the research. The number of subjects amounted to 925 female residents; however, 95 of them had medical contraindications, 453 were unable to perform the entire fitness test, 189 did not meet the age criterion, and 15 did not consent to the tests. Finally, the research group consisted of 173 older women living in SWH. The study was conducted in the common rooms of social welfare homes. The control group included women participating in a multistage project on the assessment of the physical and biological condition of elderly people in Poland from 2010 to 2016. The subjects volunteered for free tests which had been advertised in the media. However, 678 out of 1198 respondents did not meet the age criterion, and therefore, the control group consisted of 520 community-dwelling women. The examination of the control group was carried out in the Biokinetics Laboratory—the authors’ workplace.

The study inclusion criteria were (1) age 65–79 years, (2) the ability to move independently and perform a fitness test, (3) no medical contraindications, (4) written consent to participate in the study, (5) consent of the director of a social welfare home (for the residents of the institutions), and (6) independence and self-reliance (for community-dwelling women). The exclusion criteria included (1) acute injuries and infections, (2) cancer, (3) recent myocardial infarction, and (4) other medical contraindications. In order to eliminate the fluctuations in the study, all the examinations were performed in the morning, by the same researchers, and with the use of the equipment recommended by the authors of the Senior Fitness Test. For the same reasons, all the tests and measurements were conducted on the same day.

The study protocol was approved by the Senate Research Ethics Committee of the authors’ institutions (Approval Date: 2015) and complied with the ethical requirements for human experimentation in accordance with the Declaration of Helsinki. The subjects were informed about the purpose and methods of the research, all the procedures, and experimental risk. All persons who declared participation in the study signed an informed and voluntary consent document. This research is part of a large study that was retrospectively registered on the ISRCTN platform as ISRCTN 18225729. 

### 2.2. Data and Measurements 

A short questionnaire was used to collect data on age, education, place of residence, number of children, physical activity, alcohol consumption, smoking, and health self-assessment. Height (Ht) and body weight (WT) were measured with an accuracy of 0.1 cm and 0.1 kg, respectively, using an electronic balance with an integrated SECA 764 digital stadiometer (Seca GmbH & Co. KG. Germany). 

Functional fitness was assessed using the Senior Fitness Test [14]. The test is intended for the elderly and consists of six fitness tests to evaluate the strength and flexibility of the upper and lower body, endurance, motor coordination, and balance. The following tests were applied to assess fitness: 

Thirty-second chair stand—the result of this test is the number of elbow flexions for women with a weight of 5 pounds (2.27 kg) over 30 seconds. The test is used to evaluate the strength of the upper body;

Arm curl—the result of the test is the number of times the subject stands up from the chair in 30 seconds. The test is used to assess the strength of the lower body;

Back scratch—the result of the test is a distance between the fingers measured with an accuracy of 0.5 cm. The test is used to assess the flexibility of the upper body;

Chair sit and reach—the result of the test is a distance between the fingers and toes measured with an accuracy of 0.5 cm. This test is used to evaluate the flexibility of the lower body;

Eight-foot up and go—the result of the test is the time needed to cover the distance of 2.44 m, measured with an accuracy of one-hundredth of a second. The test is used to assess motor coordination and balance. 

### 2.3. Statistical Analysis 

The frequency of occurrence (%) was calculated for qualitative (categorised) data. The results of all measurements are presented as mean ± standard deviation (Mean ± SD). The normality of variables distribution was checked with the Shapiro–Wilk test. The results of functional fitness tests were compared with the standards for the elderly population in Poland developed by Ignasiak et al. [15]. For all tests, the following categories of results were adopted: ‘below the norm’—results below the 25th percentile in the 30-second chair stand, arm curl, chair sit and reach, back scratch, and for the results above the 75th percentile in the 8-foot up-and-go test; all other results of functional fitness were considered ‘normal’. The cut-off points for reduced functional fitness are shown in Table 1. 

The differences between the residents of social welfare homes and community-dwelling women were assessed using the Mann–Whitney U test for quantitative variables and Pearson’s χ^2^ test for categorised variables. Statistical significance of the results was adopted at *p* < 0.05. All analyses were carried out using TIBCO Statistica^®^ 13.3.0 (StatSoft, Krakow, Poland).

## 3. Results 

The characteristics of the subjects are presented in Table 2. With the exception of the frequency of alcohol consumption, all parameters significantly differentiated both groups of women (*p* < 0.05). Compared with older community-dwelling women, female residents of social welfare homes were on average 2 years older; had lower body weight, height, and body mass index (BMI); mostly came from smaller cities; almost every third had no children. Overall, 66.6% of them had primary and vocational education, while among the community-dwelling women, as many as 88.7% had at least secondary education. Compared with the women from the control group, the residents of social welfare homes more often reported smoking, poor health, and lack of physical activity. 

The results of functional fitness tests indicate that the majority of women living in social welfare homes had significantly reduced physical fitness. Depending on the test category of Senior Fitness Test, from 62.4% to even 87.3% of the residents did not achieve the results typical for the population of elderly people in Poland [15].

Due to the two-year difference between the average age of women from the two groups, the differences between somatic features and functional fitness were also compared in the following age groups: 65–69 years, 70–74 years, and 75–79 years (Table 3). Although there were no differences in terms of somatic features in the youngest and oldest age groups, all other differences between women living in social welfare homes and community-dwelling women were confirmed, especially in the results of functional fitness. In the 30-second chair stand and arm curl test, the residents of social welfare homes had approximately 35–40% fewer repetitions than the control group. In the test assessing flexibility, the mean results obtained by the residents of social welfare homes in each age group did not reach the positive values characteristic of community-dwelling women. The results of the 8-foot up-and-go test indicate that, regardless of age, the walking speed of women living in social welfare homes was two times slower than the women from the control group.

## 4. Discussion 

### 4.1. Key Results and Interpretation 

The first aim of the study was to assess the sociodemographic and lifestyle differences between the female residents of social welfare homes and community-dwelling older women. Almost one-third of the residents of social welfare homes had no children, and a large percentage had one or two children. In the group of community-dwelling women, only a small percentage of the subjects had one child, while the vast majority of women had one or two children. The lack of children or difficulty in taking care of parents by few children was one of the reasons why elderly, sick, and helpless people lived in institutions. The low level of education of the female residents of social welfare homes was characteristic—almost half of them had only primary education, compared with community-dwelling older women, most of whom had secondary or higher education. The relationship between the level of awareness in the context of a healthy lifestyle with economic status and the level of education is repeatedly emphasised [16]. The level of education also has an influence on the process of ageing and physical activity undertaken by seniors [7,17]. This has also been shown by our study. A relatively high percentage of the residents of social welfare homes smoked cigarettes and did not undertake any physical activity. For comparison, among the community-dwelling women, only about 5% smoked cigarettes, and more than half undertook physical activity. 

The lower level of physical activity of the residents of social welfare homes could also be related to the place of living. Most of the physical activities undertaken by the elderly are related to housework and daily activities. People living in their own homes must independently carry out activities, such as shopping, preparing meals, cleaning, and washing. Some seniors spend their free time caring for their grandchildren. Older women are more involved in household chores than men [18]. The residents of social welfare homes do not carry out all these household duties. Daily activities performed by the residents of social welfare homes are very limited. The staff members of the institutions perform most of the daily work, and therefore, the activity of the residents is low. Seniors are merely dressed and undressed, wash, and go to the canteen for a meal on their own. The low daily activity of the residents of social welfare homes has been confirmed by studies conducted by other authors [10,19,20]. Spending time in social welfare homes was most often associated with a sedentary lifestyle [10]. According to Fisher et al. [19], living in social welfare homes was connected with the low level of physical activity, which even decreased with the age of seniors. Barber et al. [20] studied the daily activity of the residents of social welfare homes using an accelerometer for seven days. The results of the research showed that the level of physical activity of the residents was very low—they spent 79% of the day sitting. [21]. Further research is needed to analyse and assess the impact of low levels of physical activity on the functional fitness of social welfare home residents more accurately.

The subjective assessment of health by seniors living in social welfare homes was significantly lower, compared with community-dwelling elderly. Almost one-third of the residents of social welfare homes assessed their health as bad, compared with a few percent of community-dwelling women. Poor health is often the reason why older people move to social welfare homes. Older, lonely women suffering from chronic diseases often live in these institutions [22,23]. 

The second goal of the study was to evaluate the functional physical fitness of the residents of social welfare homes and community-dwelling older women. The test results indicate poor functional physical fitness of the former. The results of all Senior Fitness Test trials performed in the group of the residents of social welfare homes turned out to be significantly lower, compared with community-dwelling women. Most of the residents did not meet the range of standards developed for the Polish population [15]. The test for coordination and dynamic balance, in which more than 87% of women did not reach the normal range, produced the worst results. The mean results of the 8-foot up-and-go test conducted in the female residents of social welfare homes in each age group were above the 90th percentile, i.e., they did not even fall within the broad norms [15]. This proves a high risk of falls and their consequences, primarily health related but also economic, connected with hospitalisation and treatment. Therefore, it is necessary to introduce simple daily exercises aimed at improving the dynamic balance of the subjects. The average results of the remaining tests conducted in the group of the residents in individual age groups were below the range of norms developed for the Polish population, i.e., within the 25–75th percentile. Except for the lower body strength test conducted in the age group of 65–69, the mean results of the tests evaluating lower body flexibility and lower body strength were below the 10th percentile, which means that they were also outside the broad norm. These tests, to a greater extent, involve the musculoskeletal system of the lower extremities and the trunk rather than the upper extremities. The outcomes obtained by community-dwelling women were much more favourable. The average results of all the tests in all age groups were within the range of Polish norms. 

In the last 10 years, in addition to Poland, standards for the Senior Fitness Test have been developed by several other European countries, such as Norway, Portugal, and Germany [24,25,26]. Despite some differences, such as lower scores for the norms of flexibility tests in the Portuguese norms and slightly higher scores for these tests in the German norms, the level of functional fitness of the general European population does not differ significantly from the level of fitness of the central and eastern European population. The results of our study obtained by the residents of social welfare homes also did not fall within the scope of other European standards. Therefore, we can conclude that it is not the geographical differences and the place of residence that determine the level of seniors’ functional fitness but the living environment, i.e., staying in a social welfare home reduces the level of fitness and aerobic capacity. 

Social welfare homes should ensure the optimal quality of life for their residents and provide them with the so-called active process of ageing [27]. In Poland, according to the Regulation of the Minister of Labour and Social Policy of 23.08.2012 on social welfare homes, with the changes introduced by the Regulation of the Minister of Labour and Social Policy of 17.01.2018, support activities should enable the participation in occupational therapy, improvement of fitness, and activation of seniors. Each home is obliged to have a therapy and rehabilitation room [28,29]. According to the regulation, residents of social welfare homes ought to participate in occupational therapy and physical activity classes. The results of our study indicate a very low level of functional fitness of the female residents of social welfare homes, which may suggest that physical activity classes are not properly conducted. This assumption has been confirmed by other authors. Cross-sectional studies conducted in German social welfare homes showed that the residents were not adequately instructed on how to perform exercises, with less than half of them participating in the activities [30]. The results of other studies confirmed that older community-dwelling women were more physically fit than women living in social welfare homes [10,12]. Seniors living in social welfare homes showed a faster regression in physical fitness than community-dwelling elderly of the same age, which suggests that institutionalisation actually entails a marked decline in physical activity [12]. 

The results of our study indicate a faster regression of physical fitness in residents of social welfare homes. This assumption is reinforced by significant differences noted in the tests performed in all age groups of the women. The suggestion is also supported by the results of the studies conducted in the group of community-dwelling elderly women and those living in social welfare homes, most of which were similar to the results of our study, except for flexibility of the lower limbs in women aged 70–75, where no significant difference was found [10]. It seems that the lack of significant differences, in this case, can result from the regular use of the musculoskeletal system of the lower extremities in the activities of daily living or greater activity of the subjects, and this indicates the importance of the appropriately selected programmes for seniors. 

Low scores obtained by the residents of social welfare homes in our study in relation to the Polish reference standards indicate the need to activate the subjects. The factors considered in the study—age, somatic components, lifestyle, adaptation to physical activity, and health condition—should be considered when developing and implementing physical activity programmes for seniors living in social welfare homes, in order to maintain or even improve their physical fitness which is as an important condition for being self-reliant in the process of ageing. It is also worth considering to what extent the residents of social welfare homes can be involved in everyday activities. 

### 4.2. Limitations 

The study has some limitations. Firstly, only women were included in the research. Men live shorter than women but are characterised by a greater level of physical activity. Moreover, fewer men than women live in social welfare homes. Perhaps, this is due to the higher male mortality, which makes more older women be single and choose or have to live in a social welfare home. Therefore, it is worth undertaking further research that also includes older men. The next limitation may stem from the fact that the correlation between the place of residence and the level of physical fitness of seniors may be reversed. Perhaps, it is the low physical fitness and reduced self-reliance in daily life which makes older people live in social welfare homes. Finally, the physical activity of older women in each study group and the dietary routine were also not taken into account. Therefore, further research is needed to determine the level of physical activity in seniors and its impact on functional fitness.

## 5. Conclusions

The level of education, number of children, physical activity, and the habit of smoking differentiate female residents of social welfare homes and older community-dwelling women. The residents of social welfare homes more often show unhealthy behaviours. 

The functional physical fitness of the female residents of social welfare homes is lower than those still residing in the communities.

Keeping daily physical activity is important for physical fitness among seniors living in social welfare homes, as well as among community dwellers. Additional exercise programmes are very helpful for them, as seniors living in social welfare homes lack physical activity.

## Figures and Tables

**Table 1:** Cut-off points for reduced functional fitness in women assessed using the Senior Fitness Test battery (according to Ignasiak et al. (2020) [15]).

Age (Years)	65–69	70–74	75–79
30-Second chair stand test (no. of reps)	<12	<12	<11
Arm curl test (no. of reps)	<15	<14	<13
Chair sit-and-reach test (cm)	<0	<0	<−1
Back scratch test (cm)	<−9	<−10	<−13
8-Foot up-and-go test (s)	>6.8	>7.3	>8.4

Note: The cut-off points were established based on the research results achieved among 1188–1192 women aged 65–69; 744–746 women aged 70–74; 487–489 women aged 75–79 (in various functional fitness trials there were small fluctuations in the number of participants).

**Table 2:** Descriptive characteristics of study participants, mean ± standard deviation and frequency of occurrence (%), U Mann–Whitney test, and χ^2^ test.

	Women Living in Social Welfare Homes-SWH (n = 173)	Community Dwelling Women -no SWH (n = 520)	*p*
Age (years)	72.1 ± 4.6	70.1 ± 4.2	<0.001
Height (cm)	154.8 ± 6.9	157.5 ± 6	<0.001
Weight (kg)	65.9 ± 14.3	71.1 ± 12.1	<0.001
BMI (kg/m^2^)	27.4 ± 5.4	28.6 ± 4.7	0.005
Education			<0.001
basic	48.1%	3.5%	
vocational	18.5%	7.8%	
secondary	26.6%	49.2%	
higher	6.9%	39.5%	
Place of residence			<0.001
village	14.5%	36.0%	
small town	55.5%	36.0%	
big city	30.0%	28.0%	
Number of children			<0.001
0	31.5%	7.1%	
1–2	42.0%	77.4%	
3 and more	26.6%	15.4%	
Taking up physical activity			0.032
no	50.9%	41.5%	
yes	49.1%	58.5%	
Smoking			<0.001
no	75.7%	94.8%	
yes	24.3%	5.2%	
Alcohol consumption			0.137
no	80.6%	81.8%	
sometimes	5.6%	8.7%	
yes	13.9%	9.5%	
Subjective health condition assessment			<0.001
bad	29.2%	3.7%	
good	70.8%	96.3%	
30-Second chair stand test (no. of reps)	9.5 ± 3.3	15.8 ± 3.9	<0.001
below the norm for Polish seniors	74.6%	13.5%	
Arm curl test (no. of reps)	12.3 ± 3.8	19.4 ± 4.5	<0.001
below the norm for Polish seniors	62.4%	8.7%	
Chair sit-and-reach test (cm)	−10.4 ± 12.5	3.6 ± 8	<0.001
below the norm for Polish seniors	63.6%	18.1%	
Back scratch test (cm)	−17.2 ± 13.9	−3.2 ± 8.2	<0.001
below the norm for Polish seniors	65.3%	17.9%	
8-Foot up-and-go test (s)	13.5 ± 6.3	6.3 ± 1.1	<0.001
below the norm for Polish seniors	87.3%	16.5%	

BMI—body mass index. Note: ‘below the norm’ for Polish seniors means the percentage of the participants with scores lower than the cut-off points in low functional fitness, developed by Ignasiak et al. (2020) (Table 1).

**Table 3:** Differences between women living in social welfare homes (SWH) and community-dwelling women (no SWH), mean ± standard deviation and Mann–Whitney U test.

	65–69 Years			70–74 Years			75–79 Years		
	SWH	no SWH	*p*	SWH	no SWH	*p*	SWH	no SWH	*p*
	N = 60	N = 265		N = 52	N = 165		N = 61	N = 85	
Age (years)	66.9 ± 1.6	66.7 ± 1.4	0.696	72 ± 1.4	71.8 ± 1.5	0.338	77.3 ± 1.3	77 ± 1.3	0.058
Height (cm)	156.5 ± 7.6	158.4 ± 5.8	0.113	153.4 ± 7.3	156.7 ± 5.8	0.003	154.4 ± 5.5	156.4 ± 6.3	0.141
Weight (kg)	67.4 ± 15.7	70.7 ± 11.6	0.129	64.3 ± 15.7	72.1 ± 12.7	0.003	65.8 ± 11.3	70.1 ± 12.3	0.054
BMI (kg/m^2^)	27.4 ± 5.6	27.8 ± 5.6	0.241	27.2 ± 6.2	29 ± 5.9	0.016	27.6 ± 4.6	28.2 ± 5.5	0.183
30-Second chair stand test (no. of reps)	10.4 ± 3.4	16.6 ± 3.9	<0.001	9.2 ± 2.9	15.4 ± 3.5	<0.001	8.9 ± 3.3	14.1 ± 3.9	<0.001
Arm curl test (no. of reps)	13.3 ± 3.3	20.1 ± 4.4	<0.001	12 ± 4.1	19 ± 4.4	<0.001	11.7 ± 4	17.4 ± 3.9	<0.001
Chair sit-and-reach test (cm)	−11 ± 13.7	4.6 ± 8.6	<0.001	−10.6 ± 11.8	2.8 ± 7.6	<0.001	−9.6 ± 12	1.2 ± 7	<0.001
Back Scratch Test (cm)	−15.8 ± 12.3	−2.1 ± 7.6	<0.001	−16.1 ± 13	−3.6 ± 8.7	<0.001	−19.5 ± 15.9	−5.1 ± 8.1	<0.001
8-Foot up-and-go test (s)	11.5 ± 4.6	6.0 ± 1.0	<0.001	14 ± 6.5	6.5 ± 1.1	<0.001	15.1 ± 7.2	6.7 ± 1.2	<0.001
